# Reliability of a tool for measuring theory of planned behaviour constructs for use in evaluating research use in policymaking

**DOI:** 10.1186/1478-4505-9-29

**Published:** 2011-06-24

**Authors:** Jennifer A Boyko, John N Lavis, Maureen Dobbins, Nathan M Souza

**Affiliations:** 1 Health Research Methodology Program, McMaster University, 1280 Main St. West, CRL-209, Hamilton, ON L8S 4K1, Canada; 2 McMaster Health Forum, Centre for Health Economics and Policy Analysis, Department of Clinical Epidemiology and Biostatistics, and Department of Political Science, McMaster University, 1280 Main St. West, CRL-209, Hamilton, ON L8S 4K1, Canada; 3 School of Nursing, McMaster University, 1200 Main St. West, Hamilton, ON L8N 3Z5, Canada

## Abstract

**Background:**

Although measures of knowledge translation and exchange (KTE) effectiveness based on the theory of planned behavior (TPB) have been used among patients and providers, no measure has been developed for use among health system policymakers and stakeholders. A tool that measures the intention to use research evidence in policymaking could assist researchers in evaluating the effectiveness of KTE strategies that aim to support evidence-informed health system decision-making. Therefore, we developed a 15-item tool to measure four TPB constructs (intention, attitude, subjective norm and perceived control) and assessed its face validity through key informant interviews.

**Methods:**

We carried out a reliability study to assess the tool's internal consistency and test-retest reliability. Our study sample consisted of 62 policymakers and stakeholders that participated in deliberative dialogues. We assessed internal consistency using Cronbach's alpha and generalizability (G) coefficients, and we assessed test-retest reliability by calculating Pearson correlation coefficients (*r*) and G coefficients for each construct and the tool overall.

**Results:**

The internal consistency of items within each construct was good with alpha ranging from 0.68 to alpha = 0.89. G-coefficients were lower for a single administration (G = 0.34 to G = 0.73) than for the average of two administrations (G = 0.79 to G = 0.89). Test-retest reliability coefficients for the constructs ranged from *r *= 0.26 to *r *= 0.77 and from G = 0.31 to G = 0.62 for a single administration, and from G = 0.47 to G = 0.86 for the average of two administrations. Test-retest reliability of the tool using G theory was moderate (G = 0.5) when we generalized across a single observation, but became strong (G = 0.9) when we averaged across both administrations.

**Conclusion:**

This study provides preliminary evidence for the reliability of a tool that can be used to measure TPB constructs in relation to research use in policymaking. Our findings suggest that the tool should be administered on more than one occasion when the intervention promotes an initial 'spike' in enthusiasm for using research evidence (as it seemed to do in this case with deliberative dialogues). The findings from this study will be used to modify the tool and inform further psychometric testing following different KTE interventions.

## Background

Knowledge translation and exchange (KTE) interventions in the health system aim to address the gap between research evidence and behaviour/practice/policy by targeting patients, providers, and managers or policymakers [1]. The Canadian Institutes of Health Research describe KTE as a process involving "the synthesis, dissemination, exchange and ethically sound application of knowledge to improve the health of Canadians, provide more effective health services and products and strengthen the healthcare system" [2]. In order to determine whether a KTE intervention improves the use of research evidence it must be evaluated [3]. Although models are available to help guide the development and subsequent evaluation of KTE interventions that aim to support evidence-informed policymaking [4,5], there is a lack of valid and reliable measurement tools for evaluating changes in the use of research evidence among health system policymakers and stakeholders (i.e., individuals representing specific groups or organizations that have an interest in a specific policy issue or in the system more generally).

**Figure 1:**
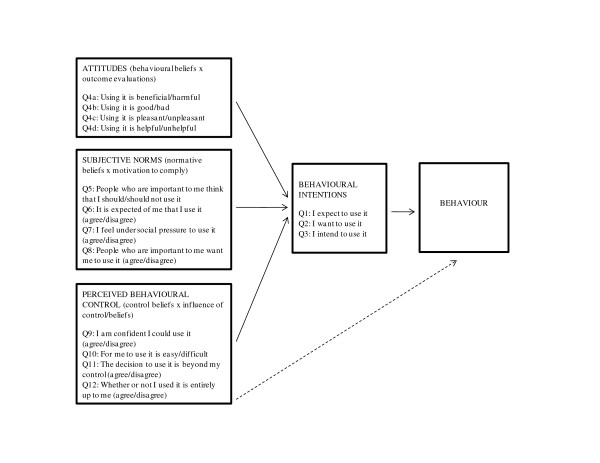
**TPB constructs, relationships between them and how the constructs relate to the 15-item questionnaire**.

One way to evaluate the effectiveness of KTE interventions, which has been used in other fields, is to measure intention to perform a behaviour (e.g., intention to use research evidence in policymaking) before and after exposure to an intervention. In the absence of objective measures of change (e.g., observable behaviours), subjective measures (e.g., one's intention to perform a behaviour), such as those derived from social cognition theories, have been used to assess changes in health-related behaviours of individuals and practice-related behaviours of providers [6,7]. The theory of planned behaviour (TPB) in particular has been extensively used and tested in the health sector. The theory focuses on intention to engage in a particular behaviour (in this case the intention to use research evidence in policymaking) and three variables (attitude, subjective norm, and perceived behavioural control) that the theory suggests will predict the intention to perform the behaviour [8,9]. An overview of the TPB constructs and the relationships among them is provided in Figure 1.

The efficacy of the TPB in predicting individual health-related behaviours has been demonstrated in several systematic reviews [10-12]. For example, a meta-analytic review in the psychology field demonstrated that the TPB can explain 20% of the variance in prospective measures of the actual behaviour of individuals [12]. There is also evidence to support using the TPB to predict the use of research evidence (e.g., clinical practice guideline implementation) in the practice of healthcare professionals [13-15]. For example, a systematic review focused on the relationship between intention and behaviour among clinicians found that the proportion of the variance in clinicians' behaviour explained by intention was similar in magnitude to that found in the broader literature [14]. Since the TPB has been useful in predicting behaviour among health professionals in terms of patient care, it may also be useful in evaluating behaviour among other professional groups involved in more system-level decision-making, such as policymakers and stakeholders [16]. The efficacy of other social cognition models (e.g., social cognitive theory and theory of interpersonal behaviour) in predicting behavior among health professionals has been less well established [7,15].

Application of the TPB in any new context requires a tool to measure the variables related to the behaviour of interest and its correlations and, like any other measurement tool, it should demonstrate evidence of psychometric properties, such as validity and reliability [17,18]. The field of psychometrics is concerned with the study of measurement instruments, and reliability and validity are central concepts to the field. In order to contribute to an understanding about whether the TPB is a useful theory for measuring change in the behaviours of policymakers and stakeholders in the health system, we carried out a reliability study to explore the internal consistency and test-retest reliability of a tool that we developed for measuring the TPB constructs that may predict the use of research evidence in policymaking.

We iteratively constructed the measurement tool using a manual designed to guide the development of valid and reliable measures of key TPB constructs for use with healthcare professionals [8,9]. As a first step, we reviewed the research literature and consulted with researchers with expertise in KTE and the TPB in order to define the target behaviour (i.e., using research evidence in policymaking) and salient beliefs associated with this behaviour. The relevant research literature included a systematic review of the attributes of processes that combine different forms of evidence to produce health system guidance [19]. Next, we defined our target behavior in terms of its target (i.e., research evidence of the type discussed at the stakeholder dialogue), action (using the research evidence), and context/time (i.e., in what I will say in a brief, advocate for or decide) [9]. Although there are various dimensions of research use in system-level decision-making such as when it is used (e.g., problem definition or options framing), how it is used (e.g., instrumental or conceptual uses), and whether and how it is documented (e.g., methods report or citation of the most compelling article), we did not specify a dimension of research evidence use because we wanted the tool to capture the full range of potential uses.

Based on the literature and input from experts we devised questions that reflected each of the key TPB constructs (Figure 1). The tool (additional file 1) includes twelve questions (15 items) that reflect each of the key TPB constructs: generalized intention (items 1 to 3); attitude (items 4a to 4d); subjective norm (items 5 to 8); and, perceived behavioural control (items 9 to 12). We constructed a short questionnaire (i.e., a subset of items were selected from the larger recommended questionnaire) because the aim of our tool is to measure behavioural intention, not to identify specific beliefs that contribute to predictor variables or to assess the influence of predictor variables in order to design an intervention to modify predictors [9]. A seven point Likert scale is used for each question. A note is included at the beginning of the tool that asks respondents to answer the questions by keeping in mind a scenario when they may have been asked to brief or provide advice to policymakers or when they may have been personally involved in a policy debate or decision-making. This is an important aspect of the tool and is meant to remind policymakers and stakeholders about the many ways in which they may contribute to the policymaking process. Although some stakeholders may not perceive themselves as being a contributor to the policymaking process, it is expected that the tool be used with policymakers and stakeholders that are in a position to champion efforts (i.e., through policy briefings, advocacy efforts or decision-making processes) to address health systems issues.

Although the manual used to guide the development of the tool assures readers that a 15-item tool designed in accordance with the manual's guidelines will represent the TPB constructs that predict intention, we decided to assess face validity in two ways. First, the tool was reviewed by four individuals with expertise in the TPB and in KTE targeted at health policymakers and stakeholders (chosen from among those we consulted in devising the items) to ensure the items appropriately assess the four TPB constructs that together predict the target behaviour (i.e., the use of research evidence in policymaking). Second, we pilot-tested the first draft of the measure following a KTE intervention that included 28 participants and that aimed to support evidence-informed action to achieve health goals in low and middle-income countries. Following the pilot test, we asked the participants in the pilot to identify any items that were difficult to understand or confusing. Based on feedback from experts and 'pilot testers,' minor revisions were made to the tool.

## Methods

### Sample and setting

Our reliability study sample consisted of 62 policymakers and stakeholders who participated in one of four stakeholder dialogues organized by the McMaster Health Forum in 2009 http://www.mcmasterhealthforum.ca/[20]. These dialogues bring various players involved in the policymaking process together (e.g., policymakers and managers from a variety of sectors, professionals, citizens/consumers and researchers) to learn from each other and from the research evidence about a specific problematic issue, options for addressing it, as well as implementation considerations. Each dialogue typically includes 16-22 policymakers and stakeholders who are chosen based their ability to articulate the views and experiences of particular groups, engage with individuals with different views and experiences, and champion efforts to address the issue following the dialogue. The study protocol was approved by the Hamilton Health Sciences/Faculty of Health Sciences Research Ethics Board (Project #: 09-402).

### Tool administration

We administered the tool to participants in the four stakeholder dialogues, both immediately following each event (T1) and again two weeks later (T2). T1 was done as part of the McMaster Health Forum's standard evaluation procedures. The two-week time interval was chosen in order to reduce the likelihood that participants underwent any contextual changes (e.g., change of job) that may have affected their responses [21,22]. At T1, we provided participants with a package that included a personalized cover letter, project summary and tool. Participants were asked to return their completed tools at the conclusion of the dialogue (in keeping with McMaster Health Forum evaluation procedures). At T2 we sent participants who completed the tool at T1 the same package, as well as a stamped, addressed envelope so that the completed tool could be returned easily. One week after T2, all non-responders received a telephone or email reminder and were sent another full-package if requested. Two weeks after T2, non-responders received another telephone or email reminder (depending on their preferred mode of communication) and sent another full package if requested. Given the short time period between test adminstrations and given that participants were not likely to be available on the telephone (i.e., the nature of their roles do not lend themselves to being in the office or they may have administrative assistants who take their calls) email reminders were a reasonable alternative to postal reminders.

### Internal consistency

There was an insufficient number of ratings per item to perform a factor analysis (i.e., we had less than the well accepted convention that there should be at least 10 cases/participants for each item being tested). Therefore, we estimated each construct's internal consistency using alpha and G coefficients. More specifically, we assessed the extent to which the tool measures each of the four constructs in a consistent way because it is expected that scores on these items correlate highly with each other [9]. Alpha coefficients were calculated for T1 and T2 scores and then averaged. G coefficients were calculated using T1 scores and the average of T1 and T2 scores. Both of these coefficients are values between zero and one that represent the extent to which items consistently differentiate between participants. It has been recommended that internal consistency coefficients should be above 0.7, but not higher than 0.9 [21-23].

### Test-retest reliability

We used two methods to quantify the tool's test-retest reliability. First, a classical test theory coefficient known as the Pearson correlation coefficient (*r*) was calculated for each construct's composite score using T1 and T2 data. Pearson's *r *is a value between -1.0 and 1.0 that describes the linear relationship between two observations [21] such that a correlation of 1.0 represents a perfect positive linear relationship and a correlation of -1.0 represents a perfect negative linear relationship [24]. Although the intraclass correlation coefficient is commonly used to assess reliability, *r *is a suitable alternative when data are used pairwise (i.e., T1 and T2 are matched) [21]. Second, we used G theory, which recognizes that measures have multiple sources of error [21], to determine whether item scores could be generalized across different administrations of the tool. We calculated G coefficients that took into account variance arising from time (i.e., variance due to participants and items was held constant). G-string_II software was used to calculate the G coefficients. Although there is no agreed upon level for test-retest reliability coefficients, a minimum reliability of 0.7 has been recommended when the test is used for research [24].

## Results

The response rate for the T1 administration of the tool was 85% (n = 53). Among those that completed the tool at T1, 43 were sent the T2 administration of the tool. (We only included individuals for the test-retest study that lived/worked in North America due to the short time interval between administrations.) The response rate for the T2 administration of the tool was 86% (n = 37). The mean time interval between participants completing the T1 and T2 administrations of the tool was approximately 30 days, with an inter-quartile range of 10 days, which we determined to be acceptable because we hypothesized that intention to use research is a fairly stable feature of an individual's mindset. We used the date the T2 tool was received (mail, fax or email) as a proxy of the date completed because we could not know when it was actually completed. Subjects described themselves according to the following role categories: public policymaker (n = 4), representative of another stakeholder group (n = 4), manager (n = 5), staff/member of a civil society group/community-based NGO or health professional association/group (n = 10), researcher (n = 4) and other (e.g., educator, administrator) (n = 10). Subjects had been in their current position for an average of 8.4 years. Some participants did not complete all of the items, leaving 3.6% of item values missing.

### Internal consistency

Table 1 illustrates that, within each construct, alpha ranged from alpha = 0.68 to alpha = 0.89, while G coefficients ranged from G = 0.34 to G = 0.73 for a single administration and from G = 0.79 to G = 0.89 for the average of two administrations.

**Table 1:** Internal consistency reliability coefficients

Construct	alpha^a^	G^b^
Behavioural intention	0.89	G_1 = 0.73, G_2 = 0.89
Attitude	0.73	G_1 = 0.42, G_2 = 0.85
Subjective norm	0.79	G_1 = 0.49, G_2 = 0.83
Perceived behavioural control	0.68	G_1 = 0.34, G_2 = 0.75

^a^T1 and T2 scores were averaged for each item prior to being submitted to the internal consistency analysis.

^b^Coefficients were calculated using T1 scores (G_1) and the average of T1 and T2 scores (G_2).

### Test-retest reliability

As illustrated in Table 2 the test-retest reliability *r *coefficients for each construct were: behavioural intention (*r *= 0.29); attitude (*r *= 0.67), subjective norm (*r *= 0.77) and perceived behavioural control (*r *= 0.70). Using G theory the test-retest coefficients for each construct ranged from G = 0.31 to G = 0.62 for a single administration and from G = 0.47 to G = 0.86 for the average of two administrations. The test-retest reliability of the 15-item tool using G theory was moderate (G = 0.5) when we generalized across a single observation, but became quite strong (G = 0.9) when we averaged across both administrations of the tool.

**Table 2:** Test-retest reliability coefficients

Construct	*r*	G^b, c^
Behavioural intention	0.89	G_1 = 0.31, G_2 = 0.47
Attitude	0.73	G_1 = 0.38, G_2 = 0.80
Subjective norm	0.79	G_1 = 0.62, G_2 = 0.86
Perceived behavioural control	0.68	G_1 = 0.52, G_2 = 0.82
Overall tool	--	G_1 = 0.51, G_2 = 0.89

^b^Coefficients were calculated using T1 scores (G_1) and the average of T1 and T2 scores (G_2).

^c^Facets: differentiation (P); generalizability (T); fixed (I)

## Discussion

### Key findings

Two key findings have emerged from our study. First, the internal consistency of items within each construct is good (0.7 ≤ alpha ≤ 0.9). Although we found no other published study that reported the internal consistency of a tool based on the theory of planned behavior to measure intention to use research evidence among policymakers and stakeholders, others have reported similar results for tools that measure intention among patient groups [17,25,27].

Second, although test-retest reliability was moderate, the more robust estimates of test-retest reliability arising from G analyses conducted on the average of both administrations suggest that, under conditions like those created by a deliberative dialogue, the tool should be administered on two occasions (at least until the tool can be revised and/or testing of a revised administration schedule or a revised tool can be carried out) in order to have confidence that the data collected by this tool consistently discriminates between respondents. In our study, the first administration of the tool immediately followed a KTE intervention that promoted an initial 'spike' in enthusiasm for using research evidence among participants and higher measures of intention on the first administration of the tool as compared to the second (i.e., intention may be a stable feature of an individual's mindset once the initial surge in enthusiasm has passed). While other research applying G theory to explore the reliability of TPB constructs found good test-retest reliability when the constructs of attitude and intention were measured on only one occasion, the same research also concluded that reliability of the other constructs (i.e., perceived behavioural control, subjective norm) would be strengthened if they were measured on more than one occasion [28]. As the current study demonstrates some good and promising psychometric properties, it seems reasonable that this tool be used in practice as a way to assess the use of research evidence in policymaking. However, in light of the G analyses that suggest a limitation of the tool is that it should be administered at least twice in order to obtain reliable data when the intervention engenders an initial surge of enthusiasm for using research evidence, further development and testing of the tool is required.

### Strengths and limitations

This study has a number of strengths. First, we developed the tool using the TPB [8], which is a well established theoretical framework, and we also used a guide developed for health services researchers to construct questionnaires using the theory [9]. This guide has been used by others to develop tools for measuring the TPB constructs [29-32]. Second, the tool is a new application of the TPB that can be used to measure one KTE outcome, namely intention to use research evidence among health system policymakers and other stakeholders. Valid and reliable tools for assessing KTE effectiveness, such as the one developed and evaluated in this study, will allow more implementation research to be carried out in order to determine what KTE strategies are effective in specific policy contexts. Third, G theory was used to assess the reliability of the tool. This is noteworthy because although tools have been developed based on the TPB to measure healthcare professionals' intention to perform certain behaviours, the psychometric properties of these tools have not always been assessed beyond face and content validity [33]. Moreover, when stability and internal consistency are explored, classical test theory has been used, which does not account for the full range of error that can be part of measurements [33,34].

The development and testing of the tool also had limitations. First, the small sample size prevented an assessment of construct validity, an important psychometric property. A measure's construct validity encompasses the idea that conceptually related items should be related statistically [35] and is usually assessed using a criterion measure or factor analysis. Since no known measures exist which measure TPB constructs to assess the use of research evidence in policymaking, criterion-related validity (or statistically comparing new measures with existing known valid measures, administered in parallel)[35] was not feasible in this study. Although there are many 'rules' for sufficient sample sizes in factor analysis, generally there should be 3-10 items per case (i.e., participant) or a minimum of 100 cases in the sample. A sufficient sample size would have allowed the theory underlying the tool to be tested using confirmatory factor analysis. Second, the measure was tested with a sample of policymakers and stakeholders who participated in one type of KTE intervention (i.e., a deliberative dialogue). As such, further research is needed to determine whether the tool is valid and reliable when used to measure TPB constructs following other types of KTE interventions (e.g., an evidence service) that involve policymakers and stakeholders.

## Conclusion

Overall, this study provides preliminary results about the reliability of a tool that can be used to measure key TPB constructs in relation to the use of research evidence in policymaking. The findings from this study will be used to modify the tool and then further psychometric testing will be carried out. Based on our findings, future psychometric testing should address three issues. First, a sufficiently large sample size should be used in order to assess construct validity through a factorial design. Item-total correlations for each construct could also be assessed to determine whether items within each construct are sufficiently related to one another [21]. Second, an assessment of criterion validity should be carried out by comparing measurements of intention to behave in a particular way to determine whether intention to use research evidence is a suitable substitute for measuring the actual behaviour. Assessments of criterion validity should also consider that evaluating change in KTE research will always be limited by fact that using intention as a proxy measure will never be a perfect substitute for measuring actual behaviour. Third, future testing should include further test-retest and internal consistency reliability tests carried out with other groups of policymakers and stakeholders exposed to different KTE interventions that aim to increase the use of evidence in policymaking. Administering the tool following a KTE intervention that does not promote an initial surge in enthusiasm for using research evidence (e.g., providing access to an evidence service) may strengthen estimates of reliability. Or, test-retest reliability could be examined after an initial surge in enthusiasm has settled (e.g., T1 might be two weeks following the intervention instead of immediately after) or without any prior intervention at all. While further development and psychometric testing is warranted, the preliminary psychometric properties of the tool suggest it is a promising KTE measurement tool.

## Competing interests

At the time of this study Jennifer Boyko was the Lead, Evidence Synthesis and Evaluation with the McMaster Health Forum. In this role she led the development of evidence briefs. She also administered, managed and analyzed brief and dialogue evaluations. John Lavis is the Director of the Master Health Forum. In this role he manages the Forum and co-chairs the Steering Committee and related sub-committees. He is also the primary facilitator for the deliberative dialogues.

## Authors' contributions

JNL conceived of the idea for this study. JAB led the development of the study protocol, implementation of the research, analysis, interpretation of findings and manuscript preparation. JNL and MD provided valuable guidance to carry out this study and contributed ideas and insights. NMS carried out interviews as part of the face validity testing and provided support in the use of G theory. All authors read and approved the final manuscript.
